# Saving Time Saves Lives: Optimizing Radio Dispatching in Out-of-Hospital Cardiac Arrests

**DOI:** 10.7759/cureus.70257

**Published:** 2024-09-26

**Authors:** Terry J Riddle, Melinda Lane, Robert Fair, Raylon Bryant, Skipper Bertrand, Justin Northeim, Kari Northeim

**Affiliations:** 1 Emergency Medical Services (EMS), Parker County Hospital District, Weatherford, USA; 2 Medical Direction, Emergency Medicine, Beacon Emergency Services Team Emergency Medical Services (BEST EMS), Grapevine, USA; 3 Population and Community Health, University of North Texas Health Science Center, Fort Worth, USA

**Keywords:** emergency dispatch, emergency medical service, emergency medical services (ems), flight paramedicine, ohca, paramedic emergency medical services, prehospital, pre-hospital emergency, prehospital emergency medicine, prehospital first responders

## Abstract

Objective

Activation of emergency medical services (EMS) through radio dispatching in the United States of America is the established first component in the link of the Chain of Survival. However, little is known about how auditory dispatch alerts operationally aid in the recognition and physical response of priority dispatch communications. This research aims to determine if a modification in radio alerting procedures will elicit a reduction in chute times for first responders.

Methods

This study uses a retrospective pre-post design evaluating the impact on reaction times to cardiac arrest priority p-tones in a semi-urban/rural area. Data were collected by comparing a period of six months before and 10 months after the implementation of a system that replaced p-tones with digitized human speech notifications. The analysis of continuous data to determine statistical significance in response times (global positioning system (GPS)-measured) was conducted using the Student's t-test. For data normalization, Box-Cox transformations were utilized. The interpretation of control charts was used to assess process stability and evaluate the outcomes.

Results

Of the 16 months of continuous data and 137 case response times for priority alarms, the average response time (GPS-measured) was 29.3 seconds (M = 29.375, SD = 19.69), well below the system target time of 60 seconds. Results of the paired sample t-test show that the mean time did not differ before treatment (M = 27.86, SD = 27.213) and after treatment (M = 30.88, SD = 27.872) at the 0.05 level of significance, t(65) =.802, n = 65, p<.425. 95% CI for the mean difference: -5.384 - 12.617, r =.032. Process control charts indicated a slight reduction in the process efficiency. A secondary finding indicated that radio utilization time was reduced by five seconds due to the intervention.

Conclusion

Response times for EMS, including the characteristics of the priority p-tone and speech influence, are understudied. This case study introduced a methodology for designing chute time process improvement interventions. Process stability charts bring increased opportunities to measure and manage response times in EMS.

## Introduction

Research on out-of-hospital cardiac arrests (OHCA) has extensively explored various aspects, including compression-only cardiopulmonary resuscitation (CPR), community education, early defibrillation, and medical treatments, to improve survival rates [1]. However, achieving faster chute times and the dispatch process by emergency medical services (EMS) personnel through improved methods of dispatch notification for emergency response has received little attention compared to advancements in medical interventions. [2,3]. This gap in knowledge is particularly evident in the understudied area of radio dispatch operations and tone signaling in EMS, as well as the lack of standardization among different dispatch entities, thus indicating a potential lack of understanding of available radio alerting procedures.

Observational studies have indicated that faster EMS response times are associated with increased survival rates for witnessed OHCA, aligning with the American Heart Association's (AHA) emphasis on rapid defibrillation in the Chain of Survival [4]. As the first component of the Chain of Survival, the activation of EMS through radio dispatching plays a critical role [5]. While dispatching processes may vary, standardized steps can be identified, including call reception, location determination, assessment of nature of call, appropriate unit determination, and dispatching. Dispatch systems have evolved with technological advancements and utilize global positioning systems (GPS) integration to minimize response times based on location [6]. However, the dispatch process lacks standardization, comprehensive study, and testing to determine if the chute times of EMS units can be improved. Human reaction time studies have provided insights into the correlation between reduced response times and higher survival rates in OHCA cases, offering potential opportunities for improvement [7].

Another important aspect to consider in this study is sound disambiguation or the constant barrage of sounds in EMS. Sound disambiguation involves distinguishing between different tones or sounds to identify the nature of the emergency. In EMS, multiple tones may be used to indicate different types of emergencies, such as cardiac arrest, trauma, or medical of origin. A study by Hansen et al. found that the use of verbal dispatch instructions instead of traditional EMS tones resulted in faster response times for EMS personnel [2]. However, the effectiveness of this sound disambiguation method across various EMS settings remains uncertain and requires further research to determine the most effective approach to alerting EMS personnel to cardiac arrest emergencies.

Parker County Hospital District Emergency Medical Services (PCHD EMS, Weatherford, TX), of their own initiative, have sought to improve the dispatch process through pre-alerts, optimizing staffing, and various other self-imposed initiatives. In coordination with Beacon Emergency Services Team Emergency Medical Services (BEST EMS, Grapevine, TX) medical direction and in partnership with the University of North Texas Health Science Center (Fort Worth, TX), researchers sought to understand the lack of standardization of the dispatch process, which creates a system where each service is free to choose how to operate. This is a complete deviation from the science of medical care provided by first responders. Working within the confines of the system created by PCHD EMS, researchers attempted to find a way to optimize the dispatch process in a way that would enhance patient outcomes. The Prehospital Assessment of the Role of Adrenaline: Measuring the Effectiveness of Drug Administration in Cardiac Arrest (PARAMEDIC-2) study and the Journal of the American Heart Association have both shown that time to medically trained professional intervention in OHCA shows positive correlations with patient outcomes [8, 9]. The peer-reviewed data regarding response times in OHCA highlighted the impact that short-term differences can have on patient outcomes. Cardiac arrest tone change was determined as one of the few areas of research that potentially has the greatest impact.

## Materials and methods

The PCHD EMS utilizes two distinct methods for tracking response times for individual ambulance units once dispatched to calls for service. One method is solely based upon crew members pressing an “En route” button on the computer-aided dispatch system in the ambulance. Potential errors with this method could be introduced, including but not limited to the ambulance not physically moving, both crew members not present to respond, or accidental activation. The second method involves the use of a GPS that tracks the physical movement of the ambulance apparatus, updating every six seconds. For the purpose of this research, it was determined that using the GPS data would provide a more accurate basis that eliminates the potential for human errors. Therefore, “chute time” is defined by the GPS data that is used to track the physical reaction of ambulance personnel.

The materials and methods used in this study were designed to assess the impact of changing dispatch methods on EMS chute times in a semi-urban/rural area. This was a retrospective pre-post study, focusing on a comparison between two distinct periods: six months before and 10 months after the implementation of a new system. This system replaced traditional cardiac arrest priority p-tones with digitized human speech notifications. A power analysis was conducted to determine the sample size required to detect a 10-second or greater improvement in chute time with a significance level of 0.05 and a power of 0.80. Using an effect size of 0.5, the analysis indicated that a minimum of 64 paired samples were needed to detect the specified effect size. This calculation was based on an expected standard deviation of 20 seconds and aimed to provide sufficient power to detect a meaningful change in response times. The study encompassed 137 case response times over a total of 16 months, providing a robust dataset for analysis. Data normalization was achieved through Box-Cox transformations, ensuring the comparability and reliability of the statistical assessments. The primary statistical tool used was the Student's t-test, which evaluated the mean response times before and after the intervention to ascertain any significant changes. This method is particularly effective in comparing means from two different groups with a designed intervention.

Moreover, the study employed control charts to evaluate process stability and outcome effectiveness. Process charts with capability process (Cp) index values were used to quantitatively assess the capability of the EMS system both before and after the intervention. These charts were established with a minimum of 0 seconds, a target of 60 seconds, and a maximum of 120 seconds for response times. Control charts help in distinguishing between common cause variation (natural variability inherent in the process) and special cause variation (variability due to specific, identifiable factors). The control limits are set at ±3 standard deviations from the process mean, providing a range within which the process is expected to operate if only common cause variation is present.

**Figure 1:**
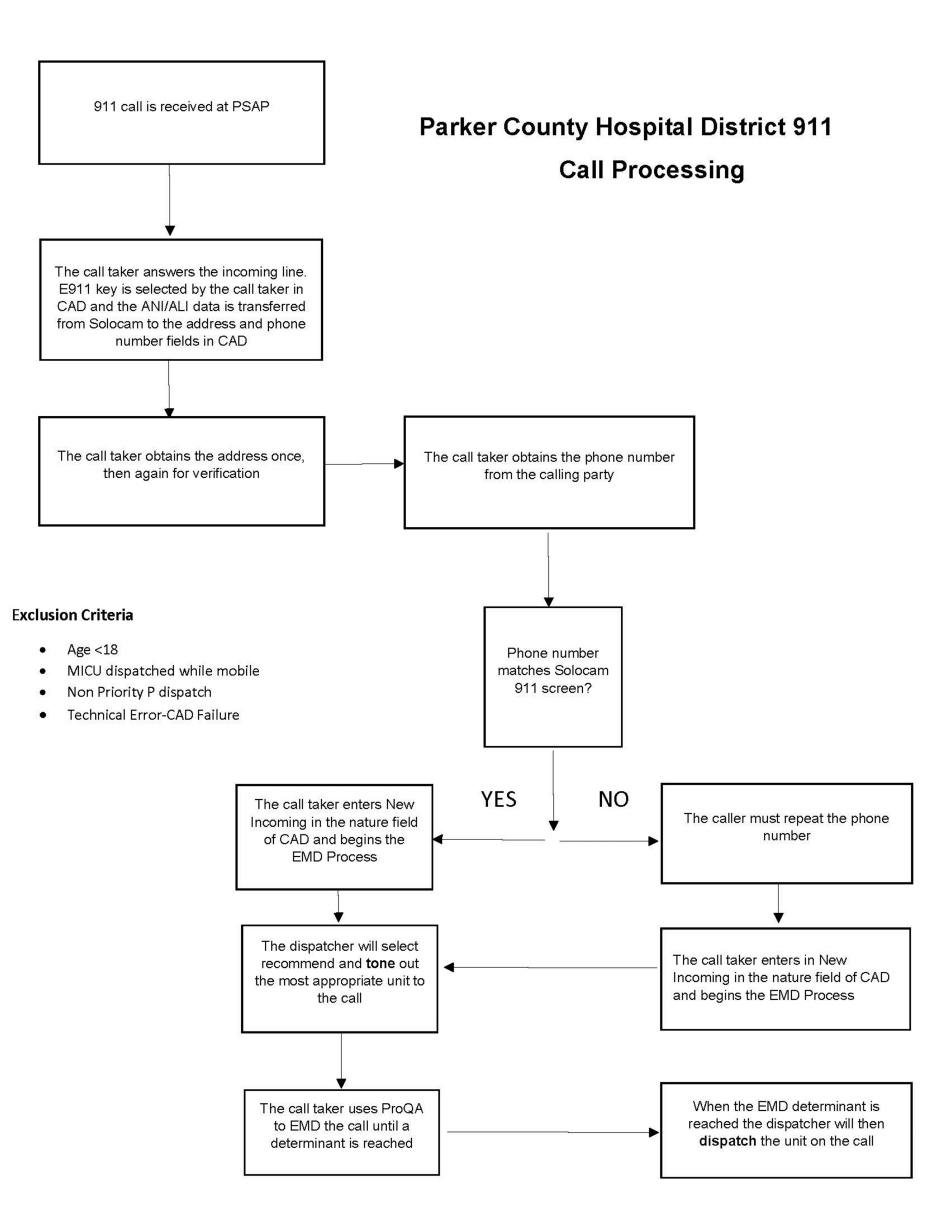
Parker County Hospital District dispatch process for all calls generated in the CAD system PSAP: Public Safety Answering Point; MICU: mobile intensive care unit; CAD: computer-aided dispatch; ANI: automatic number identification; ALI: automatic location identification; EMD: emergency medical dispatch

Figure 1 illustrates the call-taking process of certified dispatchers at PCHD EMS, from initially receiving a 911 call then following the decision tree, resulting in an ambulance unit being dispatched. 

Study setting

Case Study Area

As of the 2021 United States Census, Parker County, Texas, has a population of 156,764 that reside within 910 square miles, west of Fort Worth [10]. Parker County Hospital District established the PCHD EMS to provide an ambulance response system in 1984. The agency began utilizing computer-aided dispatch in 2016 for dispatching ambulances. In 2022, the system was dispatched to over 15,000 emergency calls, of which 225 were cardiac arrest dispatches. 

Participants, Data Collection, and Outcome Measures

Computer-aided dispatch information was obtained from the PCHD EMS for calls requiring emergency medical services during two distinct time periods. From May to December 2022, PCHD EMS implemented changes to its cardiac arrest dispatch notification system by eliminating a specific alert tone and switching to a digitized voice message only. This study utilized six months of historical data collected prior to the implementation of the changes, as well as 10 months of data following the implementation. The treatment variable includes a digitized voice message to elicit reaction times [2]. Due to this modification, the crew alerting system is simplified, distinct, and shortened.

This study analyzed group performance instead of individual performance, as EMS providers work in two-person teams that constantly rotate with variable partners and shifts. If the ambulance is out of the station when dispatched, the data are not included in the analysis, nor are any participants under the age of 18. However, all calls where the ambulance was stationary and located at the station were included [11]. Moreover, field staff paramedics were informed of the changes to the priority p cardiac arrest tones via company email prior to the initiation of the change, without specified reasoning for the tone change. Field staff were then exposed to the tone change at the onset of the beginning of their 48-hour shift.

Ethics approval was obtained through the North Texas Regional Institutional Review Board (approval number: IRBNET 2029282-1).

## Results

**Figure 2:**
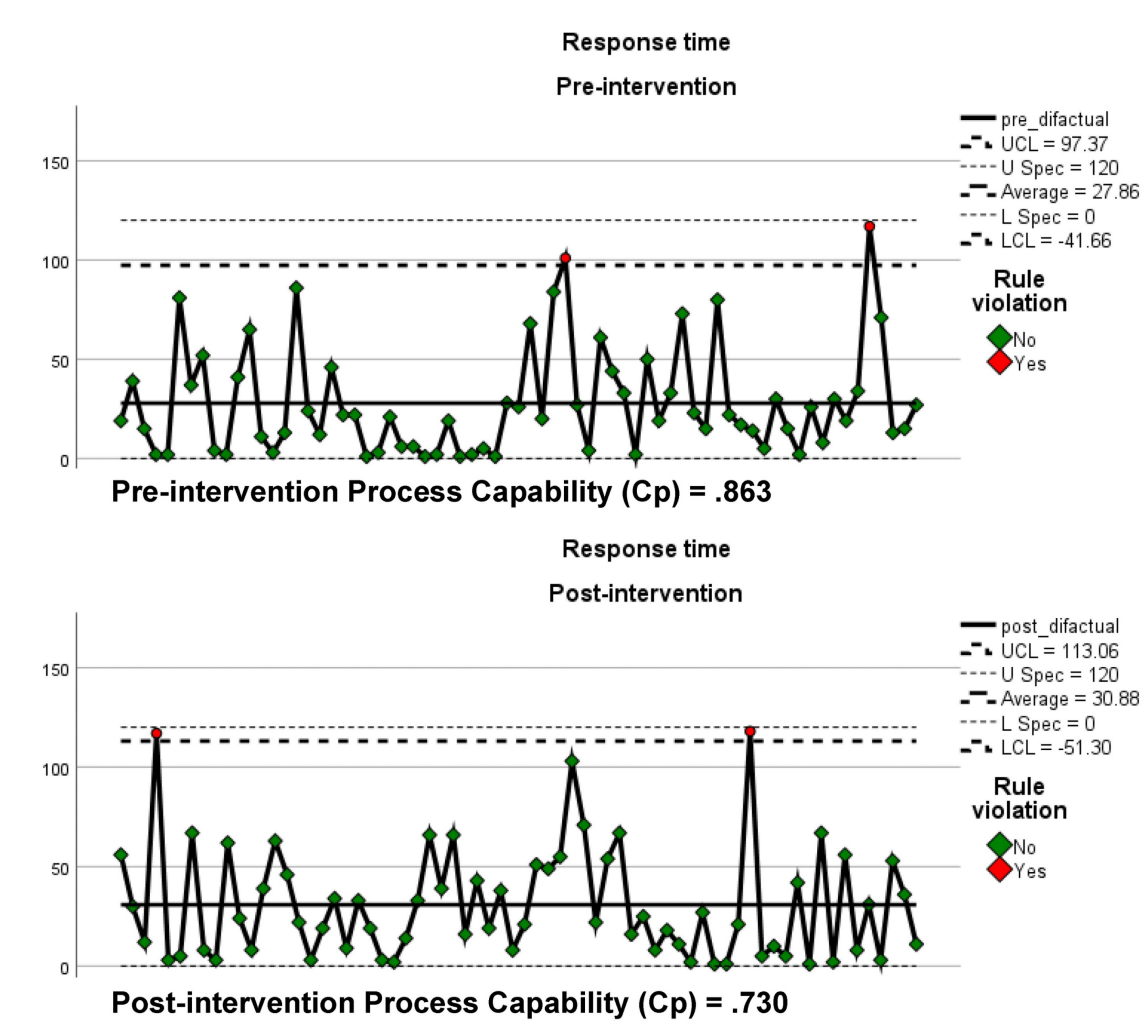
Pre- and post-intervention process control charts Cp: capability process index; UCL: upper control limit; U-spec: upper specifications of 120 seconds; L Spec: lower specifications of 0 seconds; LCL: lower control limit

Over a period of 16 months, encompassing 137 case response times, this study identified an average EMS chute time of 29.3 seconds (mean = 29.375, standard deviation = 19.69). Interpretation of the control charts indicates that the chute time process remained stable both before and after the intervention, with no significant special cause variations observed (Figure 2). However, the slight increase in variability and reduction in Cp values (0.863 pre-intervention to 0.730 post-intervention) suggest that while the process did not deteriorate significantly, the intervention did not enhance the overall capability of the system. 

A statistical analysis was conducted to assess the impact of the dispatch intervention. The results of a paired sample t-test showed no significant difference in response times before and after the intervention: the mean response time pre-intervention was 27.86 seconds (SD = 27.213), compared to a post-intervention mean of 30.88 seconds (SD = 27.872), resulting in a t(65) =.802, n = 65, p <.425. The 95% confidence interval for the mean difference spanned from -5.384 to 12.617, with a correlation coefficient of r =.032. 

## Discussion

Emphasizing the importance of efficient workflow processes in EMS, the study utilized process control charts and noted a slight decrease in process capability post-intervention [12]. This minor change does not significantly impact the effectiveness of the new dispatch method but strengthens the importance of a comprehensive approach in EMS, as advocated by Aringhieri et al. [13]. In addition, the consistent chute times under 60 seconds, aligned with National Fire Protection Association standard 1710, demonstrate the system's effectiveness [14].

**Figure 3:**
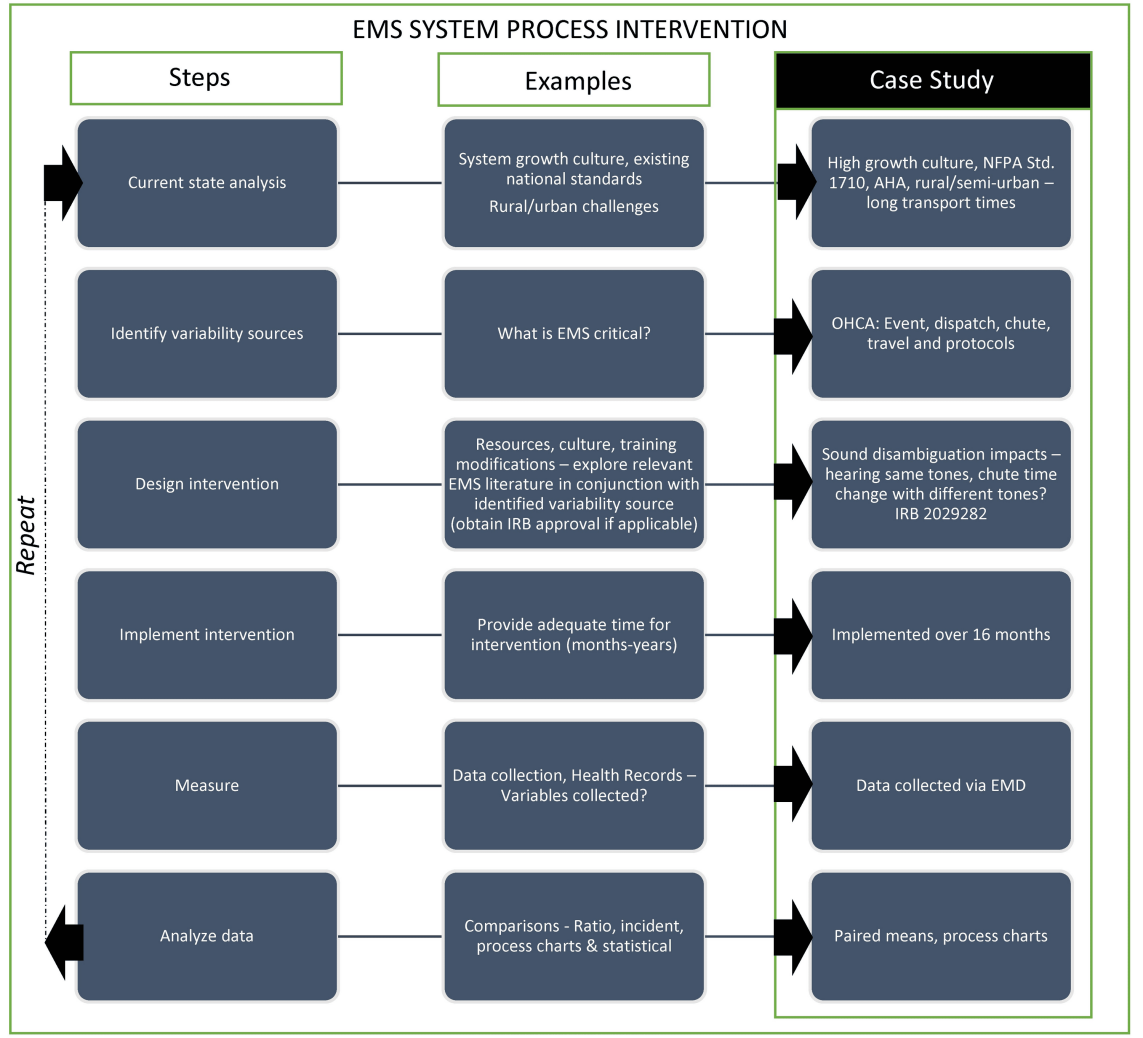
Theoretical model for reducing chute times NFPA Std: National Fire Protection Association standard; AHA: American Heart Association; EMS: emergency medical services; OHCA: out-of-hospital cardiac arrests; EMD: emergency medical dispatch This model has been developed by the authors.

Although the average chute times did not change, the intervention calls for further research in EMS dispatch processes, especially considering evolving operational challenges and technologies [15]. An added benefit of post-intervention was a reduction in radio traffic time by five seconds, potentially freeing up radio waves for other critical calls or information exchange. The reduction in radio traffic highlights added benefits that may be revealed through a scientific approach to the dispatch process. It is critical that EMS continues to seek ways in order to reduce these chute times since current research has established a correlation between response times by EMS personnel and overall survival rates of OHCA patients. During the research, a theoretical model of process intervention was developed as a template for current and future changes (Figure 3). While system status management and tiered response systems have the potential to reduce response times, station-based EMS services are limited due to the structure of the system design. Adapting and altering the dispatch process allows each service to attempt to enhance crew chute times, thus decreasing response times.

This study investigated whether changing dispatch tones could affect EMS chute times, particularly for priority p-calls. We found no significant difference in average chute times when comparing p-tones to digitized human speech, aligning with El Sayed's discussion on the complexities of EMS response times [16]. Limitations such as crew workload, schedules, and other confounders not included in this study might influence these results. In addition, the GPS has a six-second reporting delay and a limited sample size that may have influenced the results. Furthermore, PCHD EMS utilizes a “pre-alert” system designed to elicit quicker chute times by alerting the EMS crew to an incoming call prior to the unit being officially dispatched. Thus, PCHD EMS personnel are already aware of an incoming call prior to being dispatched and are given the opportunity to prepare to respond. We assert that the pre-alert process may influence outcomes more than understood. Therefore, an interpretation of this could be that departments that do not already utilize a pre-alert system could potentially show more dynamic changes in chute times when implementing a change to priority p-tones.

## Conclusions

Emergency medicine success is often predicated on the time to intervention, whether the golden hour for trauma patients, quick delivery of broad-spectrum antibiotics, or immediate cardiopulmonary resuscitation in the cardiac arrest patient. All of these interventions have been shown to reduce mortality in each case. While the majority of research and education is focused on early recognition and medical intervention, little research has been applied to the operational aspect of EMS in order to continue to reduce the time to trained provider intervention. While there was no statistically significant change in average chute times, the case study underscores the importance of examining the entire dispatch process and its impact on emergency response. Future research should focus on optimizing chute times, possibly through dispatch process modifications, and the significance of pre-alerts in EMS. This is particularly relevant for OHCA patients, where every second counts.
